# Contextual Modulation is Related to Efficiency in a Spiking Network Model of Visual Cortex

**DOI:** 10.3389/fncom.2015.00155

**Published:** 2016-01-19

**Authors:** Fariba Sharifian, Hanna Heikkinen, Ricardo Vigário, Simo Vanni

**Affiliations:** ^1^Brain Research Unit, Department of Neuroscience and Biomedical Engineering, Aalto UniversityEspoo, Finland; ^2^Aalto Neuroimaging, AMI Centre, Aalto UniversityEspoo, Finland; ^3^Clinical Neurosciences, Neurology, University of Helsinki and Helsinki University HospitalHelsinki, Finland; ^4^Department of Computer Science, Aalto UniversityEspoo, Finland; ^5^Department of Physics, Faculty of Sciences and Technology, University Nova of LisbonLisbon, Portugal

**Keywords:** spiking network model, simulation, V1, inhibition, excitation, area summation, efficiency

## Abstract

In the visual cortex, stimuli outside the classical receptive field (CRF) modulate the neural firing rate, without driving the neuron by themselves. In the primary visual cortex (V1), such contextual modulation can be parametrized with an area summation function (ASF): increasing stimulus size causes first an increase and then a decrease of firing rate before reaching an asymptote. Earlier work has reported increase of sparseness when CRF stimulation is extended to its surroundings. However, there has been no clear connection between the ASF and network efficiency. Here we aimed to investigate possible link between ASF and network efficiency. In this study, we simulated the responses of a biomimetic spiking neural network model of the visual cortex to a set of natural images. We varied the network parameters, and compared the V1 excitatory neuron spike responses to the corresponding responses predicted from earlier single neuron data from primate visual cortex. The network efficiency was quantified with firing rate (which has direct association to neural energy consumption), entropy per spike and population sparseness. All three measures together provided a clear association between the network efficiency and the ASF. The association was clear when varying the horizontal connectivity within V1, which influenced both the efficiency and the distance to ASF, DAS. Given the limitations of our biophysical model, this association is qualitative, but nevertheless suggests that an ASF-like receptive field structure can cause efficient population response.

## Introduction

**Figure 1:**
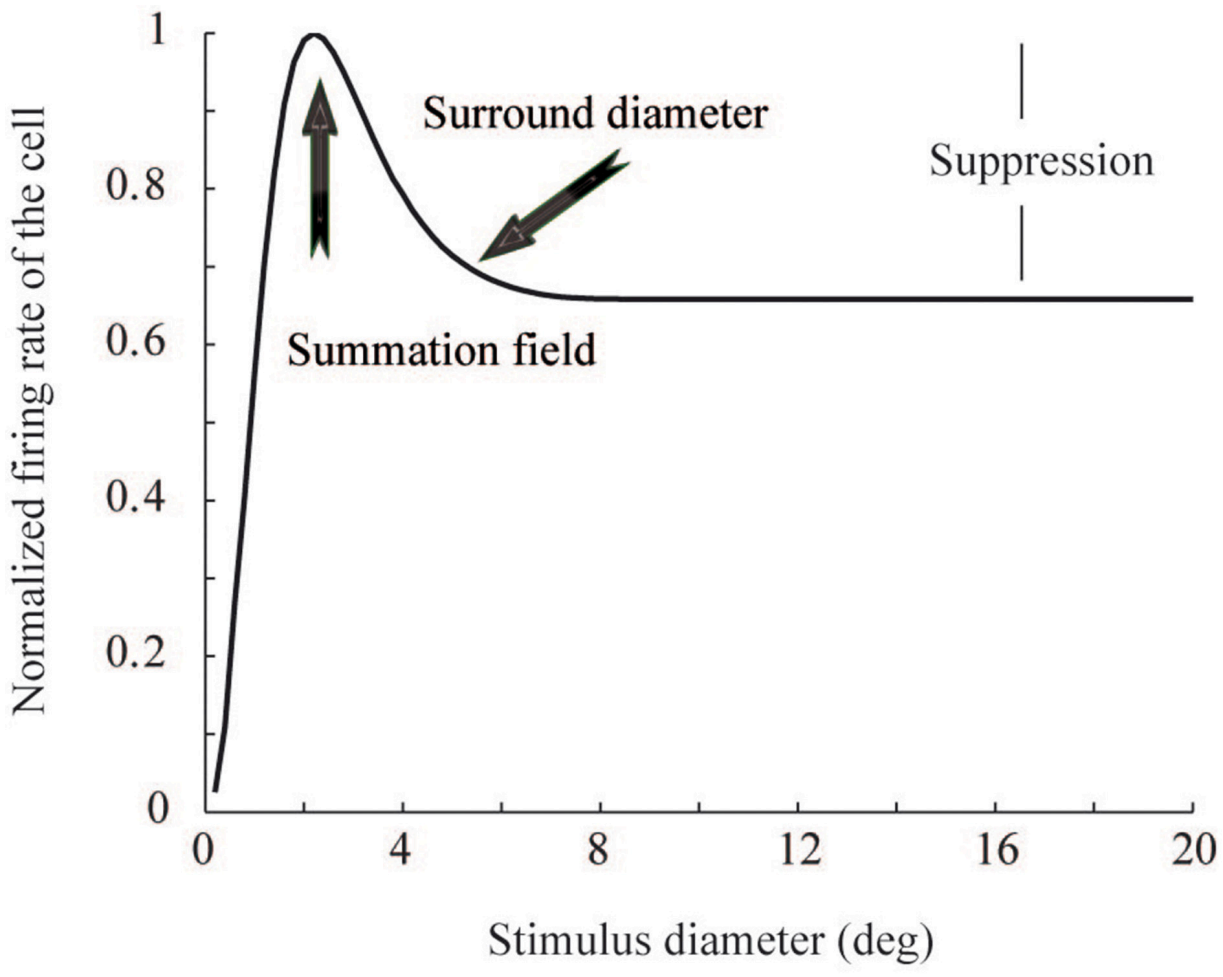
**Area summation function**. The function shows the summation field size, surround field size, and suppression strength, for a V1 neuron whose receptive field center is located at 14° eccentricity. The three parameters come from linear regression of monkey V1 data (**Figure 3**).

Contextual stimuli outside the classical receptive field (CRF) modulate the responses of visually responsive cortical neurons (Maffei and Fiorentini, 1976; Knierim and van Essen, 1992; Levitt and Lund, 1997; Sceniak et al., 1999; Li et al., 2000; Felsen et al., 2005). In the primary visual cortex (V1), contextual modulation has been studied by systematically changing the size of a circular grating stimulus centered in the cells' receptive fields (Sceniak et al., 1999; Angelucci et al., 2002; Cavanaugh et al., 2002). The typical response curve, the area summation function (ASF), starts with a sharp increase in neural firing rate, as a function of increasing stimulus diameter (Figure 1). When the stimulus diameter exceeds a certain size, called summation field, the response begins to drop, eventually reaching an asymptote. However, although widely reported, it is still not clear why such non-linear area summation is useful for biological visual signal processing. In particular, we studied whether the non-linear ASF can be related to an improvement in energy consumption and coding efficiency within the visual system.

There is no standard definition of coding efficiency, although it usually requires a specific goal of optimization in presence of a constraint. In a neural network, one definition of coding efficiency can be the information that is transmitted (as the goal) with one unit of energy (as the constraint). An early theoretical study suggested that reduction of redundancy in correlated neural activation patterns will improve coding efficiency (Barlow, 1961). Later, experimental (Baddeley et al., 1997) and theoretical (Olshausen and Field, 1996) work showed that a sparse code is expected to emerge as a result of coding efficiency. Sparse code in a network is the case where a fraction of neurons encode the signal, while the rest remain relatively silent.

Several studies have associated contextual modulation with efficient coding. In particular, Vinje and Gallant (2000) showed that stimulation outside the CRF increases population sparseness and decorrelates neural responses in the V1 of macaque monkeys. They suggested that inputs from the classical and outside CRF s together contribute to the formation of a metabolically efficient sparse code. Schwartz and Simoncelli (2001) suggested that the visual system can use knowledge about image statistics surrounding the CRF, to reduce dependencies between single cells and thus increase efficiency. In addition, in previous studies we suggested indirectly that contextual modulation increases efficiency of the population responses (Sharifian et al., 2013), as measured with fMRI. However, earlier work has not attempted to link the ASF directly to coding efficiency. Because ASF is a prominent and common receptive field property in the visual cortex, such a link would emphasize that coding efficiency may be a primary evolutionary driver for contextual modulation. Hence, we hypothesized that the ASF, which is an expression of contextual modulation at single cell level, might be directly associated with coding efficiency in visually responsive neurons (Vanni, 2012).

**Figure 2:**
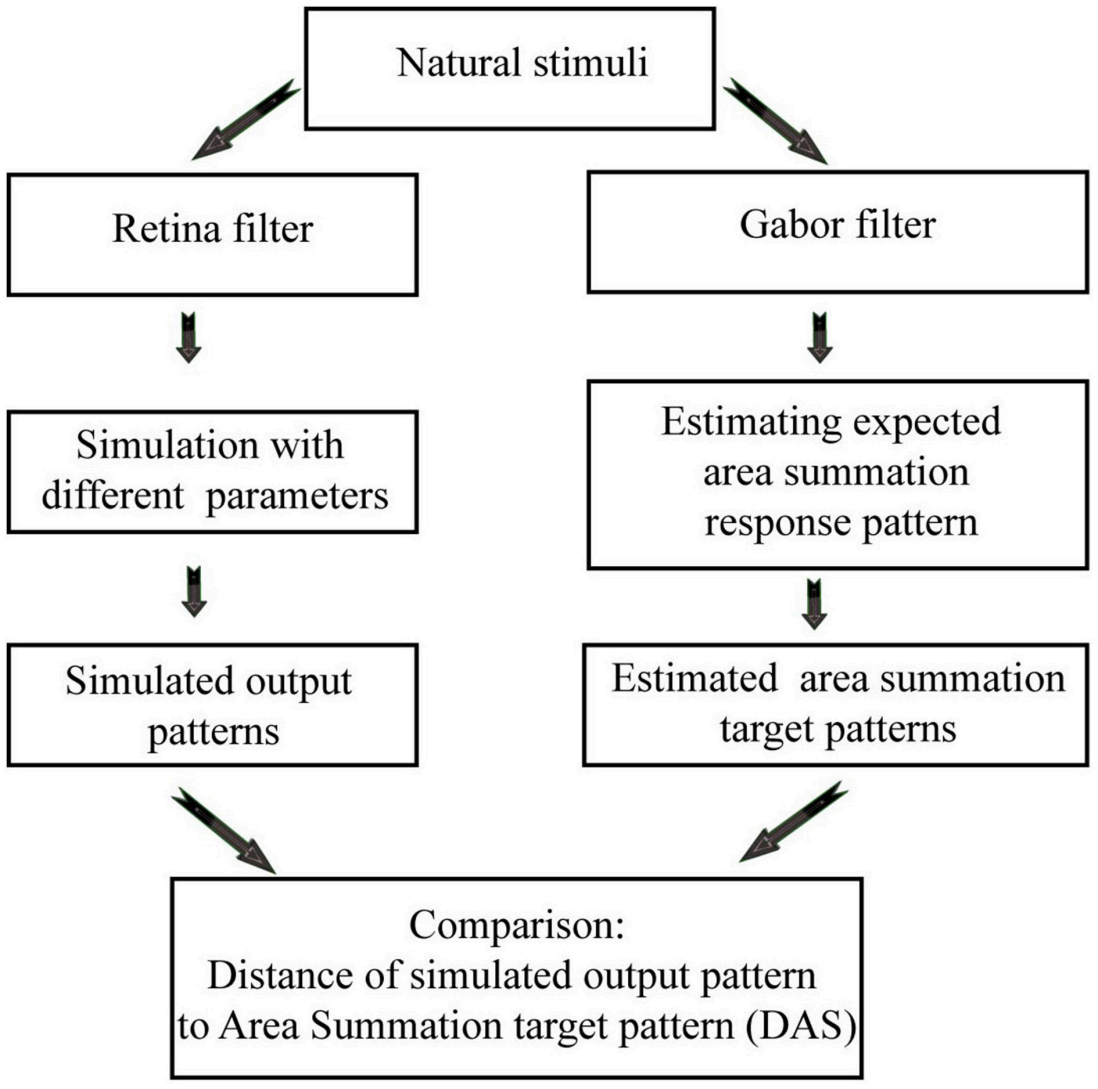
**Flowchart of the data analysis, showing how the biomimetic simulations (on the left), and area summation target patterns (on the right) are generated from the natural images**.

Cavanaugh et al. (2002) provided quantitative data from macaque cortex, as well as a mathematical description of the ASF. The ASF function, together with a biophysically meaningful (biomimetic) 2D spiking neural network simulator of the primary visual cortex (Heikkinen et al., 2015) gave us the opportunity to study quantitatively the relation between the ASF and the network efficiency of a model visual cortex. The biomimetic neural network simulator provides a powerful tool to explore the mechanisms which help the brain (population of neurons) to work more efficiently. We wanted to investigate if area summation is one of these mechanisms or not. Unlike abstract mathematical models, such biomimetic model approach supports acting much closer to biological reality and thus better explains how an efficient code might emerge in biological network. In short, we aimed to answer two questions: (i) when simulated neural activation becomes close to biological ASF, does efficiency increase? (ii) If yes, can we find parameters of the model which affect this efficiency? We simulated the response of the model network to natural images, while varying network parameters, in order to obtain outputs with varying degree of conformation with the biological reality. The network output was then compared to the predicted spike output in the visual cortex, calculated from existing ASF data of primate V1 neurons. Figure 2 shows the flowchart of the data analysis. We show here that the non-linear ASF is strongly associated with a decrease of metabolic cost of neural signal processing, as well as with an increase of entropy per spike and sparseness.

## Methods

### Experimental area summation data

Target data for our model simulations was derived from the single cell receptive field responses from Cavanaugh et al. (2002), provided by James Cavanaugh, Wyeth Bair, and Anthony Movshon. In their study, data were collected from simple and complex cells in the primary visual cortex of adult Cynomolgus monkeys and pig-tailed macaques. The neurons were stimulated with variable contrast sinusoidal gratings, presented on a gray background. From the 352 neurons in V1, 87% showed at least five spikes per second and clear CRF boundaries. Those were analyzed further. The neurons' receptive fields were centered at eccentricities between 0 and 30°. For each single neuron in a specific eccentricity (i) summation field, (ii) surround diameter, and (iii) suppression levels were recorded. Figure 1 illustrates the prototypical ASF response curve of a neuron whose receptive field center is located at 14° eccentricity. The ASF gives the neuron's response as a function of circular grating patch size. They modeled the neural response *R*(*x*) as a function of stimulus contrast and size with a relation of two Gaussian functions, Equation (1).

(1) $$\begin{array}{l} R\left(x\right)=\frac{k_{c}L_{c}\left(x\right)}{1+k_{s}L_{s}\left(x\right)}\\ L_{c}\left(x\right)={\left(\frac{2}{\sqrt{\pi }}\int_{0}^{x}e^{-{\left(y{∕}^{\prime }w_{c}\right)}^{2}}dy\right)}^{2}\\ L_{s}\left(x\right)={\left(\frac{2}{\sqrt{\pi }}\int_{0}^{x}e^{-{\left(y{∕}^{\prime }w_{s}\right)}^{2}}dy\right)}^{2} \end{array}$$

In this ASF, *x* is the diameter of circular stimulus and [*k*_*c*_, *k*_*s*_] and [*w*_*c*_, *w*_*s*_] are the gain and spatial extents of the center and surround components' coefficients, respectively. The gain coefficients (*k*_*c*_ and *k*_*s*_) are dependent on the stimulus overall contrast (Cavanaugh et al., 2002). However, we assumed fixed *k*_*c*_ and *k*_*s*_ in our natural image stimuli. The *L*_*c*_ and *L*_*s*_ are the summed squared activations of the center and surround mechanisms, respectively.

**Figure 3:**
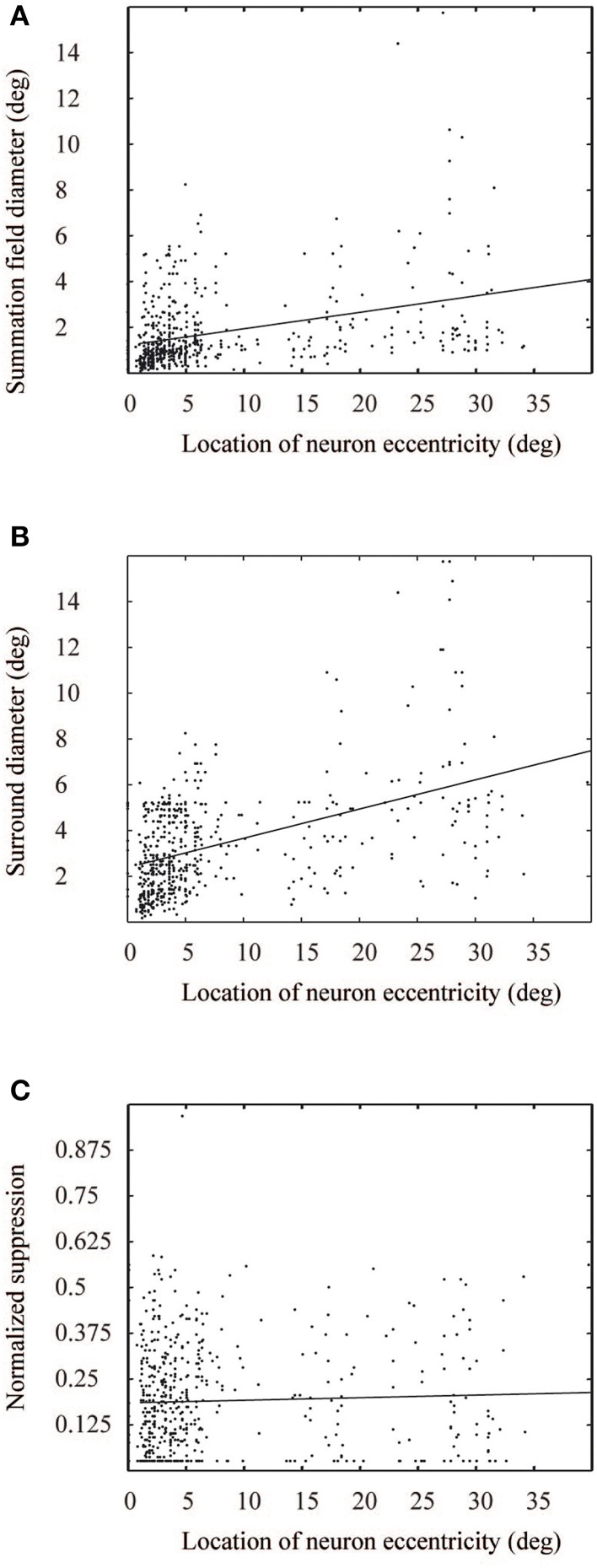
**Measured values from Cavanaugh et al. (2002), for summation field (A), surround diameter (B), and suppression level (C) as a function of eccentricity**. The solid lines show corresponding linear fits to the data points.

This model describes neural response to a grating stimulus, as a relation between excitatory and inhibitory Gaussian functions. The parameters for the Gaussian functions were estimated from the ASF data of the monkey cells (Cavanaugh et al., 2002). The ASF data comprised three parameters: summation field size, surround field size, and suppression strength (Figures 1, 3). As an approximation, linear regression was used to model each parameter as a function of eccentricity. The values for *k*_*c*_, *k*_*s*_, *w*_*c*_, and *w*_*s*_, Equation (1), were obtained from this data for each neuron, with a Nelder-Mead simplex optimization algorithm (Lagarias et al., 1998). Once the model parameters were fixed, we got estimations of *R*(*x*) for each neuron in the primary visual cortex. The response was dependent on the eccentricity of the neural receptive field center in the visual field.

### Theoretical visual neural network response based on area summation data

The area summation response [*R*(*x*), see Equation (1) and Figure 1] of the V1 neurons was next used to estimate the expected neural responses to a series of 20 grayscale natural images from the image set of van Hateren and van der Schaaf (1998).

**Figure 4:**
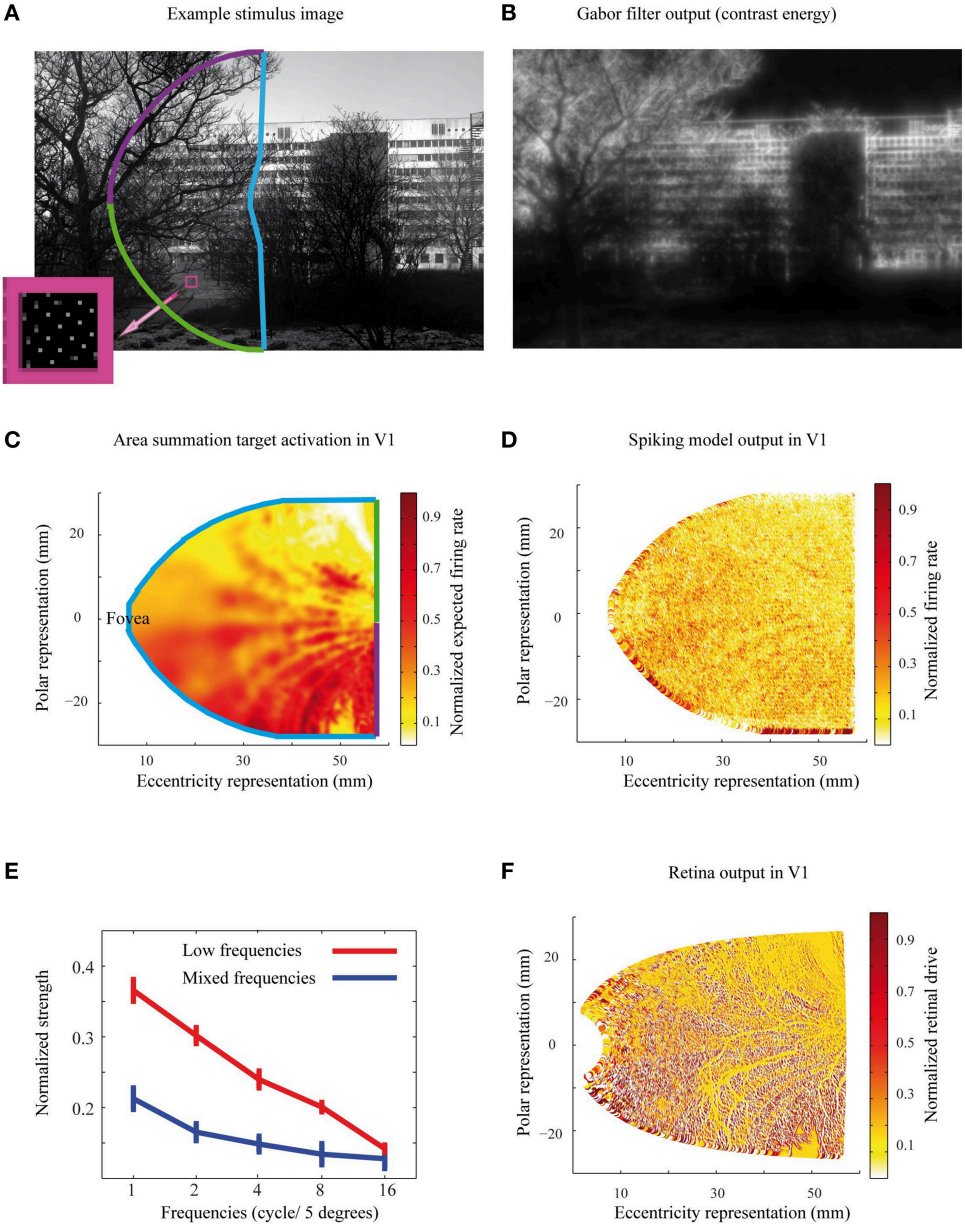
**The stimuli, predicted and simulated neural activation patterns. (A)** The selected area of an example natural image illustrates the projection of the image to the Gabor filter and ASF calculations of the left visual field. Small dots in the pink square show the cortical density of cells in the visual field. The density of cells in each eccentricity follows the human cortical magnification factor. **(B)** The contrast energy image from the same natural image as in **(A)**, built as the output of a series of Gabor filters. These contrast images are the input for estimating the target area summation patters. **(C)** The area summation target activation pattern, corresponding to the same natural image as in **(A)**. The color code of the activation pattern borders illustrates the projection of the image from the visual field to the model cortex, with the color code as in **(A)**. **(D)** Simulated output pattern for the image in **(A)**. **(E)** The normalized strength as a function of the spatial frequency contents for the natural images in Low and Mixed frequencies groups. Error bars represent the standard error of mean. **(F)** The output of the retina filter to the natural image in **(A)**, which served as an input to the spiking neural network simulations.

The ASF *R*(*x*) is determined from the neural response to circular grating stimuli of varying sizes, and is expected to predict how elements from various distances from the receptive field center contribute to the neural response. In typical experimental setup with monkeys, grating parameters (position, orientation, spatial frequency) are first optimized for each cell, when the ASF is experimentally determined. Thus, to convert this cell-specific function for general gray scale images (Figure 4A), we need to first convert the gray-scale images to contrast energy representations (Figure 4B). Then we apply the ASF filter to get the expected spatial distribution of the V1 response for a specific stimulus, i.e., the “target.” Such contrast energy image provides the spatial structure of contrast energy, with broad-band sensitivity to both orientation and spatial frequency content. Please note that this contrast energy image is comparable to the retina output for a specific stimulus and is not directly comparable with overall contrast of a grayscale image. The contrast energy was calculated by filtering the natural gray-scale images with five different Gabor filter frequencies 1, 2, 4, 8, and 16 cycles per 5° field of view (Kay et al., 2008), which capture the maximum contrast sensitivity for human vision (Hess, 2004). Here we assumed that 82 pixels cover 5° of the visual field. Each frequency comprised eight orientations with 0° and 90° offset phases, thus we had all together 5^*^8^*^2 = 80 different filters. After applying all these filters to each pixel in a natural image, we summed the normalized values of the filtered images, and used this contrast image as the input to the ASF, defined in Equation (1). The result can be seen in Figure 4B.

Next, we derived the general response function *f* (*u*,*v*), as a distance-dependent weighing function of the contrast response for a pixel at location (*u*,*v*), at distance *r* from the model neuron receptive field center, according to:

(2) $$\begin{array}{l} f\left(u,v\right)=R^{\prime }\left(2r\right)∕\pi r\\ r^{2}=u^{2}+v^{2}\\ R^{\prime }\left(x\right)=\frac{k_{c}L_{c}^{\prime }\left(x\right)\left(1+k_{s}L_{s}\left(x\right)\right)-k_{s}L_{s}^{\prime }\left(x\right)k_{c}L_{c}\left(x\right)}{{\left(1+k_{s}L_{s}\left(x\right)\right)}^{2}}\\ L_{c}^{\prime }\left(x\right)=\frac{8}{\pi }e^{-{\left(x∕w_{c}\right)}^{2}}\int_{0}^{x}e^{-{\left(y∕w_{c}\right)}^{2}}dy\\ L_{s}^{\prime }\left(x\right)=\frac{8}{\pi }e^{-{\left(x∕w_{s}\right)}^{2}}\int_{0}^{x}e^{-{\left(y∕w_{s}\right)}^{2}}dy \end{array}$$

With homogeneous distribution of pixels in the visual field, *f* (*u*,*v*) can be derived from *R*(*x*) in Equation (1). Lines 3, 4, and 5 of the Equation (2) calculate this derivative.

To obtain the relative contribution from each visual field location to a model V1 neuron's response, the derivative of the ASF response, *f* (*u*,*v*), was calculated for each location (*u*,*v*), with respect to its distance from the receptive field center of the cell. These differential weighting functions were then multiplied with the image contrast energy, separately for each point (*u*,*v*) around the model cells, and thus obtained response contributions, which were then summed over a 7° diameter area surrounding the receptive field center of each neuron. This area is clearly enough to reach the asymptote of the individual neuron's response (Figure 3). Repeating this for each model neuron, we obtained the target neural activation pattern of the primary visual cortex to each natural image (Figure 4C). These target activation patterns were then compared with the model simulation outputs (Figure 4D). The expected area summation pattern was an approximation because the variability in single cell parameters (Figure 3) was not included into the ASF model.

To control how the amount of suppression and size of the summation field is related to the network efficiency, we generated two artificial ASFs, in addition to the natural ASF. One with a larger summation field and no suppression [by multiplying *k*_*c*_ and *k*_*s*_ at Equation (1) by factors of 4 and 0.25, respectively], and the other one with a smaller summation field and stronger suppression [by multiplying *k*_*c*_ and *k*_*s*_ at Equation (1) by factors of 0.25 and 4, respectively] compared to natural ASF.

The gray-scale natural images contained highly varying features. For a simple check on how different properties of input stimuli might affect our results, we divided the input stimuli into two groups, based on the distribution of their spatial frequencies. We used the same Gabor filter frequencies as in the image preprocessing (Figure 4B, with 1, 2, 4, 8, and 16 cycles over 5° of visual field). Thus, each natural image was characterized with a 5 bin histogram of frequency values. The 20 gray-scale natural images where then assigned into two groups based on the frequency histograms, using k-means clustering approach. The low frequency group contained 10 gray-scale images, with predominantly low spatial frequencies, and the 10 images in the mixed frequencies group had closer to equal portions of high and low spatial frequencies (Figure 4E).

### Input to the biomimetic visual cortex model

The natural image input to the spiking network model of the visual cortex was first filtered through a retina model (Figure 4F). The retina model consisted of midget on- and off-cells, which both covered the left visual field from 0.5 to 27° eccentricities. Each midget cell center covered a region spatially resembling a segment of an arc, and together these segments covered the sampled visual field with no space gaps, separately for the on- and the off cells. The midget on-cells' receptive field centers were about 30% larger in diameter than those of the off-cells, and their number correspondingly smaller. The filter density followed the midget cell density in human retina (Dacey, 1993). As an approximation, each millimeter of retina covered 3.8° of visual field. This proportion was equal at all eccentricities. The retina model contained 83,934 on-type cells, with density dropping as a function of eccentricity. In an additional control simulation, we used a lower density of midget cells, corresponding to 1/5 relation of that in human retina. Correspondingly, the receptive field size was larger than in humans. This filter included 3331 on-type cells. Off-type cells were included in the retina model but were not used because there was no specificity of connections in the cortex model. Thus, the on and off-cells would have mutually canceled the net signal from retina.

The shift of midget cell inner segment positions in relation to their soma-part were compensated for the innermost 3 mm close to the fovea (Dacey, 1993). When projecting pixel images to the model retina, images were first fitted to the model visual field, and then resampled to a grid with 161,900 units per mm^2^, corresponding to the highest photoreceptor density of human retina at the fovea (Curcio et al., 1987). Next, the gray scale values (a proxy for retinal luminous flux) of the resampled grid units landing inside the midget receptive field centers were linearly summed. The center inputs were contrasted with the input from the 8-neighborhood of receptive field centers, each multiplied with value −1/8. The resulting center-surround receptive field inputs were first normalized and then transformed to relative ganglion cell firing rates with a logistic function (range from 0 to 1). Finally these relative firing rates in the retina were spatially transformed to V1 (Schwartz, 1994) using human magnification factor (Duncan and Boynton, 2003), and transformed to actual model input firing rates.

### Biomimetic spiking network simulation

We used a recursive network of exponential integrate-and-fire (EIF) neurons (Naud et al., 2008), to simulate the V1 population response to the gray scale natural images. The network was originally implemented for studying the spatial properties of neural activation and functional imaging signals on the primary visual cortex and has been described detail in Heikkinen et al. (2015). The model neurons comprised either six compartments (V1 excitatory cells) or a single-compartment (all other cells). The compartmental neurons implemented passive dendritic properties (Rall, 1962) of pyramidal neurons, adjusted to match more recent data on the decaying excitatory post-synaptic potential efficiency as a function of distance from cell soma (Williams and Stuart, 2002). Our previous study (Heikkinen et al., 2015) suggested that anisotropic landing of inputs from different origins to apical dendritic tree could underlie some of their distinct functional roles, such as the contextual modulation by visual features outside the CRF. In a set of control simulations we used the pointwise model for all neurons. The neurons received both excitatory (AMPA) and inhibitory (GABA type A) conductance. The membrane potential of the single compartment neurons and the somata of the compartmental neurons followed the EIF equation.

The simulations were run for 500 ms, with 200 ms of baseline followed by 300 ms of stationary image input. The network's output to each stimulus was evaluated as the mean spike count of each neuron between 200 and 500 ms, and the preceding 200 ms baseline was excluded from further analysis. The simulations were implemented in Brian 1.5 simulation environment (Goodman and Brette, 2008), and computations were conducted at the Finnish IT Center for Scientific Computation (CSC) Taito super cluster with maximum 448 number of parallel cores and 4GB of memory usage per core.

**Figure 5:**
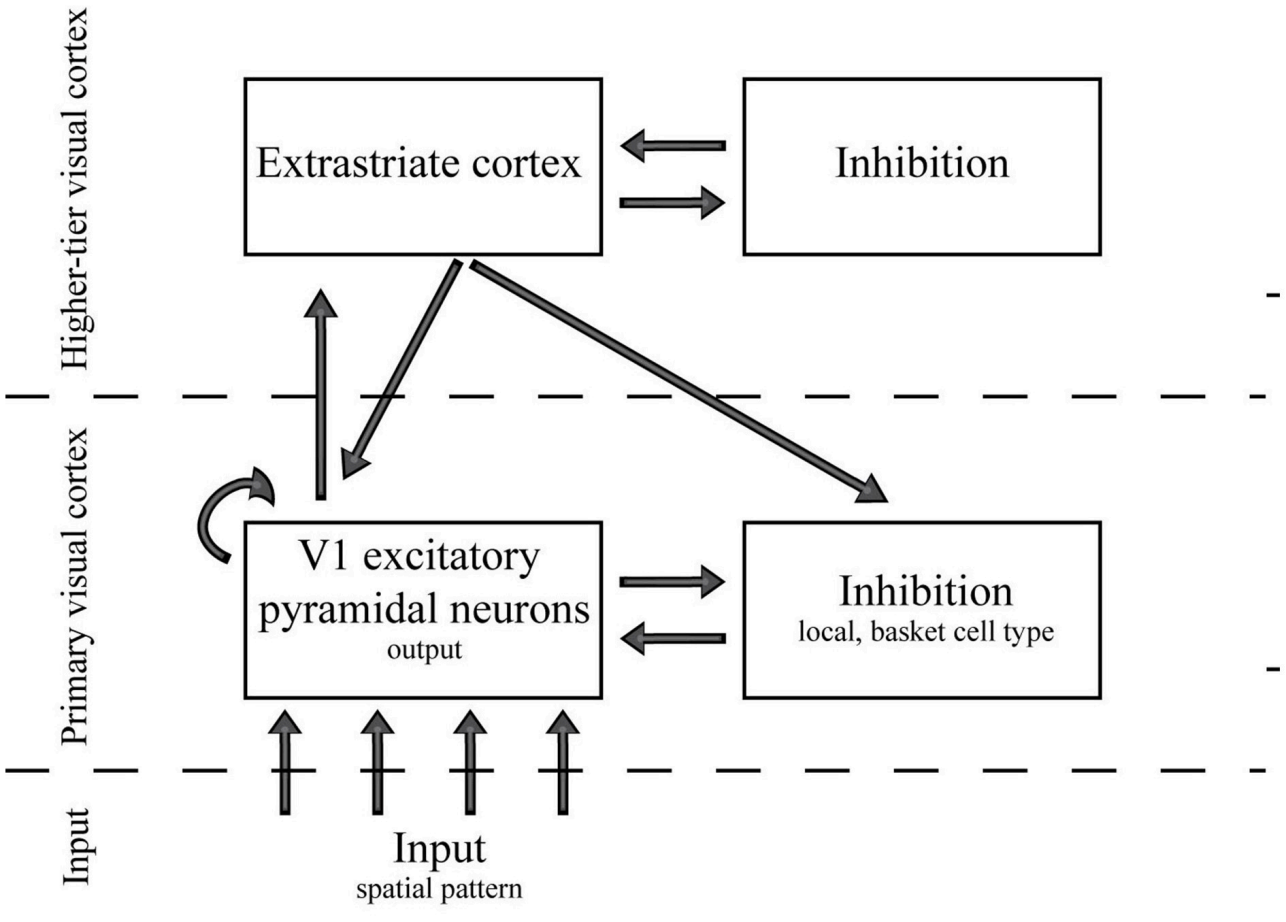
**A schematic view of the spiking network layers and connections**.

The network consisted of five different groups of neurons, in three hierarchical levels (Figure 5): Group 1: The input layer at the bottom of the hierarchy (226,206 neurons; 9014 neurons for the control simulations with low number of cells in retina), Group 2: excitatory pyramidal cells at the V1 (42,849 neurons), Group 3: inhibitory fast-spiking basket-like cells at the V1 (10,713 neurons), Group 4: excitatory extrastriate neurons (10,713 neurons), and Group 5: inhibitory extrastriate neurons (10,713 neurons). The neurons in Group 1, Group 2–3, and Group 4–5 belong to the input, primary visual cortex and higher-tier visual cortex, hierarchical level respectively. The input from LGN had a Poisson distribution with probability proportional to stimulus strength at each spatial location, and for all other neuron groups (V1 excitatory, V1 inhibitory, extrastriate excitatory, and extrastriate inhibitory) the spatial synaptic connections between two neural groups were stochastically generated. The connection probability was first determined with sparseness-parameter. Next the probability was decayed either with Gaussian function or exponentially as a function of the distance between the pre- and postsynaptic cells (Heikkinen et al., 2015). In short, the feedforward connections from lateral geniculate nucleus to excitatory V1 cells (LGN → E_V1_) were determined with Gaussian distribution with sigma of 0.23 mm along the cortical surface. The lateral connections (E_V1_ → E_V1_, E_V1_ → I_V1_, I_V1_ → E_V1_) were determined with exponential function with lambda of 1 mm, whereas the feedforward and feedback connections (E_V1_ → E_X_, E_X_ → I_X_, I_X_ → E_X_, E_X_ → E_V1_, E_X_ → I_V1_) were determined with exponential function with lambda of 3.3 mm. Above, V1 subscript denotes the primary visual cortex, X denotes the extrastriate area of the model, and E and I the excitatory and inhibitory model neurons, respectively.

In the biomimetic network, we first set an initial value of each parameter according to literature when available, and then adjusted a subset of parameters for reasonable dynamic range (see Heikkinen et al., 2015, for the motivation and details). Next, two parameter pairs which seemed to affect the results most were varied independently while other parameters were fixed. The changing parameters were (i) the number of connections between the V1 and extrastriate cortex excitatory cells (E_V1_ → E_X_, E_X_ → E_V1_, and E_X_ → I_V1)_ and (ii) the number of lateral connections in V1 between excitatory and inhibitory cells (E_V1_ → I_V1_, I_V1_ → E_V1_). These variations in the number of connections were controlled by changing a scaling factor, β, in range of 0.17-4.20 for connections probability between the V1 and extrastriate cortex and 0.1-2.45 for connection probability between V1 excitatory and V1 inhibitory cells. The scaling factor β was changed in logarithmic scale. Each pair comprised a fixed ratio of bi-directional connections parameters which scaled together. The ratios within a group were chosen to produce almost equal number of feedforward and feedback connections and strength of E_X_ → I_V1_ was tied to E_X_ → E_V1_ in order to assign 10% of the feedback to the inhibitory neurons.

The biomimetic network model was stimulated with the 20 grayscale images, filtered through the retina model. For each image, the simulations were repeated for 625 times, over which the number of connections in the network were varied to produce outputs with variable levels of biological realism. The choice of network connections followed the network optimization procedure of our previous study (Heikkinen et al., 2015), where the network output was matched to conform to both single cell electrophysiology and functional magnetic resonance imaging data, by finding a suitable set of inhibitory and feedback connections. Thus, the simulations yielded a total of 20 ^*^ 625 = 12,500 simulated response patterns that served as data points in evaluating the network efficiencgy.

**Figure 6:**
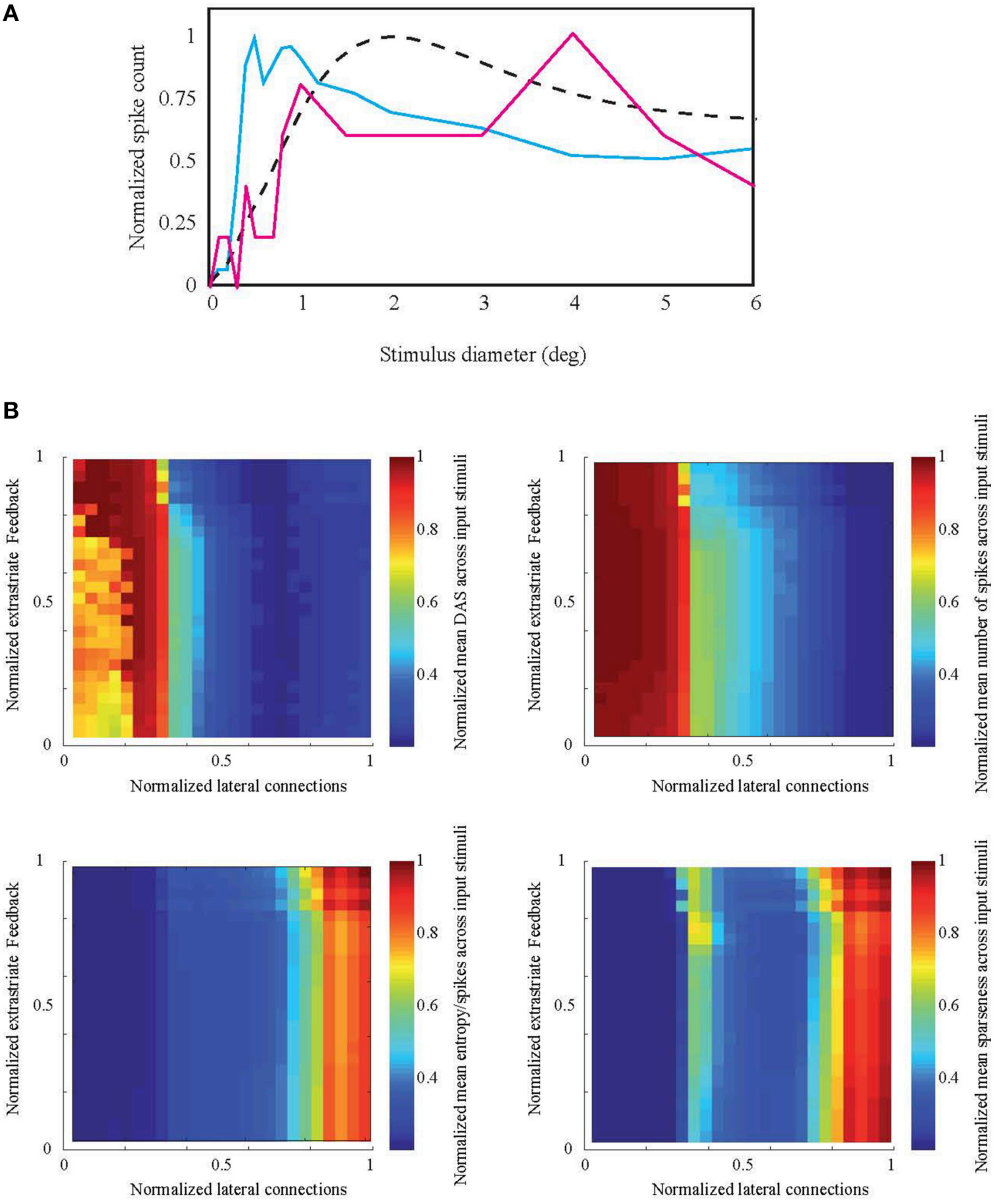
**Simulation result compared to natural ASF**. **(A)** Examples of the spiking network response associated with high (solid blue curve) and low (solid red curve) efficiency measures to an increasing stimulus patch size, for a representative model neuron located at 14° eccentricity. Response is averaged across 15 neighboring cells. The high-efficiency response is similar to ASF target at the same eccentricity (dashed curve) but with smaller summation field size. **(B)** Normalized mean distance to ASF (DAS), number of spikes, entropy per spike, and sparseness across all 20 natural images as a function of the number of V1 lateral excitatory-inhibitory and V1-extrastriate feedforward-feedback connections.

Figure 6A shows the response of a model cell located at 14° eccentricity to a circular patch stimulus. The two samples of the parameter combinations are associated with either high (solid blue curve) or low (solid red curve) efficiency. The response is averaged across 15 neighboring cells in order to avoid emerging noise to a single neuron response from stochastic nature of inputs and neural connections. For the high efficiency network, this function is similar to the ASF, although the summation field size is smaller than in the target ASF curve (dashed curve, similar to Figure 1). In contrast, the average responses of cells in the low-efficiency network do not show any clear summation peak or suppression.

### Comparison between the target activation and model simulation output

For each gray scale image, the simulated response pattern was compared to the corresponding area summation target pattern, and the comparison was repeated for all network parameter combinations. To reduce border effects, we excluded from the analysis those neurons whose receptive field centers were within 0.5° from the edge of the visual field.

To compare a simulated spiking network model response to the corresponding target pattern predicted from the neural area summation data, we first normalized both response patterns between 0 and 1, by first subtracting the minimum value from each response and then dividing it by the maximum value. The normalization was done on each network parameter combination and stimulus image separately. We then calculated the difference (absolute distance) of the simulated and target responses (mean normalized action potential frequency) for each neuron, and summed these distances across all neurons into a single measure Distance to Area Summation (DAS):

(3) $$\begin{array}{l} DAS=\overset{n}{\underset{i=1}{\sum }}|S_{i}-T_{i}| \end{array}$$

where *n* is number of neurons in the activation patterns and *S, T* are simulated and target response, respectively.

The output of the spiking network model for each stimulus image and parameter combination was evaluated with three different efficiency metrics. Our first measure of efficiency was the energy consumption related to processing one image. Attwell and Laughlin (2001) calculated energy usage based on dephosphorylation of adenosine triphosphate (ATP) molecules as follows: 3.4 × 10^8^ ATPs / s are used on average to maintain the resting potential of a neuron. In addition, 1 × 10^8^ ATPs / s are used to maintain the resting potential of associated glial cells. A total of > 10 × 10^8^ ATPs / spike are required for firing a spike, and the associated recycling of glutamate, as well as pre- and postsynaptic housekeeping actions. Their study suggests approximately a linear link (plus a baseline) between the total number of spikes in a simulated response pattern and the energy consumption of the neuron, and thus the overall spike count was selected as a simple proxy for the relative energy consumption of the network.

Our second measure of efficiency is the entropy per spike, which describes the energy efficiency of the activation patterns. Importantly, this corresponds to the entropy over the spatial pattern of the mean spike count (for all the 40k+ cells in the model V1) and not to the entropy, over time, of single cell spike trains. To calculate the entropy per spike, spike counts of the V1 excitatory cells were first normalized by dividing by the maximal spike count. Next, we calculated the probability distribution (q) of relative spike outputs from a histogram with 256 bins.

(4) $$\begin{array}{l} \begin{array}{c} ES=E∕N_{s}\\ E=-sum\left(q\log_{2}q\right) \end{array} \end{array}$$

where *E, ES* and *N*_*s*_ are entropy, entropy per spike and total number of spikes, respectively. We could then calculate the entropy based on the *q*-values, Equation (4).

Our third measure of efficiency is the neural population sparseness (Vinje and Gallant, 2000), which reflects coding efficiency of the activation patterns. The population sparseness of a simulated response pattern was calculated as:

(5) $$\begin{array}{l} \begin{array}{c} S_{p}=\left\{1-\left[\frac{{\left(\overset{N}{\underset{n=1}{\sum }}\left(y_{n}∕N\right)\right)}^{2}}{\overset{N}{\underset{n=1}{\sum }}{\left(y_{n}\right)}^{2}∕N}\right]\right\} \end{array}∕\left[1-\frac{1}{N}\right] \end{array}$$

where y_*n*_ is the mean firing rate of neuron number *n* among *N* neurons and S_*p*_ is population sparseness. In Equation (5) smaller values indicate lower level of sparseness and larger values indicate higher level of sparseness.

## Results

We simulated the neural responses of V1 in a biomimetic recurrent spiking network model, to study the relation between the non-linear area summation properties of primate V1 neurons and efficiency of the neural activity at the population level. The network was stimulated with gray-scale images of natural scenes and the parameters of the network connectivity were varied to produce outputs with varying level of biological realism. In each simulation output, the spike counts of the cells varied in average (across different images and parameters combinations) between 11 ± 27 (mean and standard deviation of minimum spike counts over 300 ms of stimulation, across all natural images and parameter combinations) to 81 ± 18 (mean and standard deviation of maximum spike counts) with average of 42 ± 34. These values are at the high end of V1 firing rates in typical experimental paradigms (Reich et al., 2001; Yan et al., 2014). Such high rates were necessary, because *in vivo* there are much larger number of synapses than in our model, and thus in our model we need higher firing rates than *in vivo* to have enough input to neurons to keep them in dynamic range. However, we normalized the spike counts of each simulation output between 0 and 1, to be able to compare them with the corresponding target created from ASF. For each parameter set and image, the spike response of the network was evaluated for three measures which together quantify the network efficiency: (1). total spike count provided an estimate for the energy consumption of the whole system, (2). entropy per spike served as a measure of the potential information content of the spike output, and (3). population sparseness, typical to cortical networks, reflects coding efficiency in two ways. The mean firing rate in a sparse code is lower than in a dense code, saving energy. Moreover, a sparse code decorrelates the neural output and thus reduces redundancy in the population response. Sparse codes are therefore able to carry informational aspects of the input with relatively fewer spikes. These efficiency measures were analyzed against difference between simulated and predicted (ASF-like) 2D spike response patterns in the V1 cortex. A low difference between spike response patterns from simulation and prediction based on ASF indicate a high association to ASF.

Figure 6B shows the Distance to Area Summation DAS target and the efficiency measures (number of spikes, entropy per spike, and sparseness) as a function of the varied parameter space. The two-dimensions of the parameter space were the number of feedforward-feedback connections between V1 and extrastriate cortex (E_V1_ → E_X_, E_X_ → E_V1_, and E_X_ → I_V1_, see Section Biomimetic Spiking Network Simulation for neural group definitions) and the number of lateral V1 connections (E_V1_ → I_V1_ and I_V1_ → E_V1_). The connections between the excitatory cells within V1 (E_V1_ → E_V1_) were earlier found to have no explanatory power to model BOLD and spiking responses. Our results show that the lateral connections between V1 excitatory and inhibitory cells have clearly stronger effect on ASF-association compared to extrastriate feedforward-feedback connections. If the lateral connections are very silent (normalized value 0 in Figure 6 horizontal axis), distance to ASF is largest. Efficiency increases with increasing strength of lateral connections. However, the distance to DAS peaks before reaching the maximum value of lateral connections.

**Figure 7:**
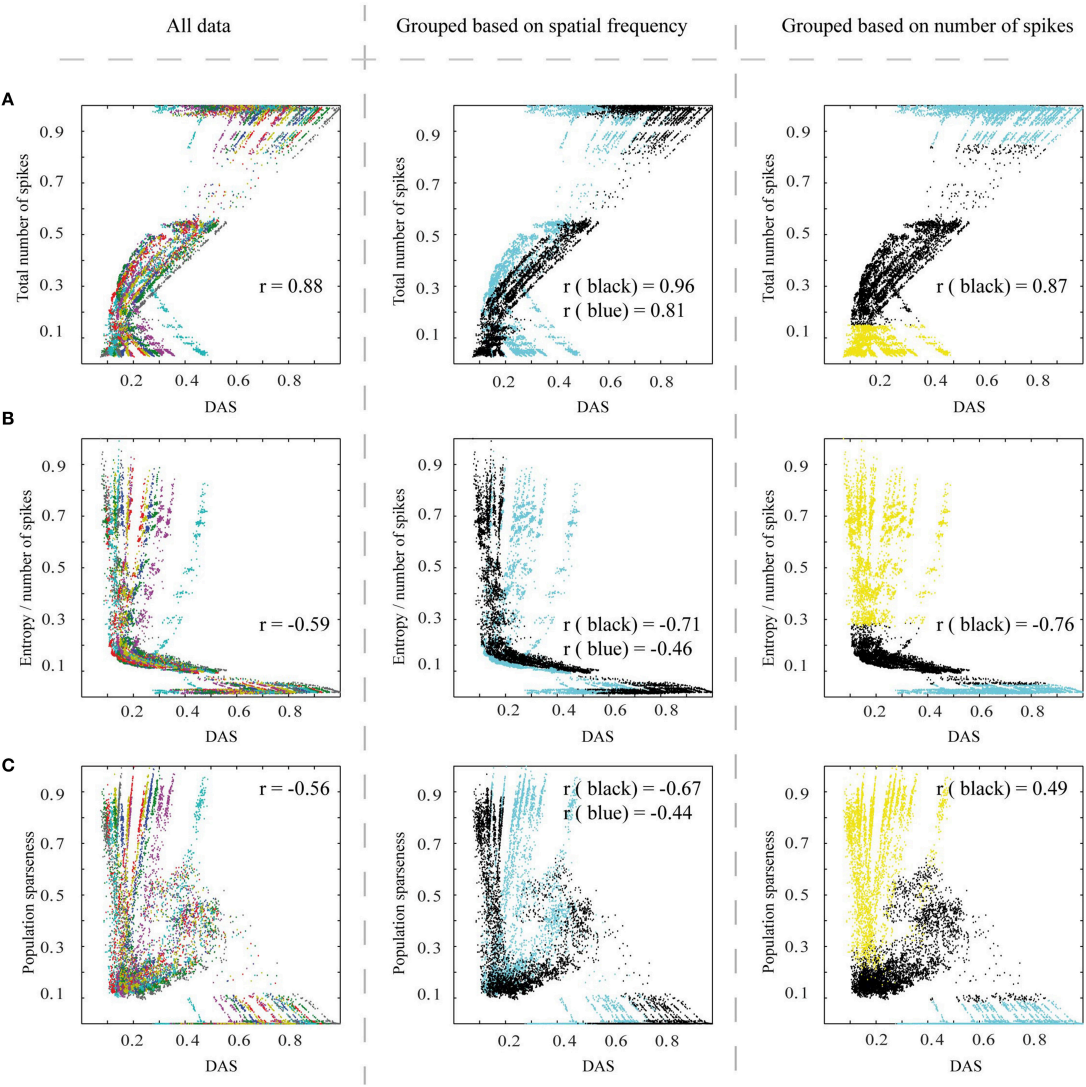
**The efficiency of the spiking network, as a function of Distance to Area Summation (DAS)**. Left column: **(A)** The relationship between the total number of spikes counted from V1 excitatory neurons (associated linearly with energy consumption) of simulated output patterns and similarity to estimated area summation target patterns. The data points in this plot represent the simulator output for the 20 images, each using 625 parameter combinations, with the data color coding for different images. 0.5° from the border, for all the patterns, were removed to avoid instability in borders. r corresponds to the correlation coefficient. **(B)** The relationship between entropy per spike of the simulated output patterns and DAS, with the same simulation output data and color coding as in **(A)**. **(C)** The relationship between neural population sparseness (0 is minimum and 1 is maximum sparseness) of simulated output patterns and DAS, with the same simulation output data and color coding as **(A)**. The middle column is the same as left column, but color coded to illustrate the division of the stimulating images to two groups, based on their spatial frequency contents (Figure 4E): black dots represent images with the low frequencies, and blue those with the mixed frequencies. Right column follows also the structure as in the left column, but color coded to indicate the data points excluded due to saturated or low-responding activation patterns (blue and yellow respectively) and the remaining data points (black).

Figure 7 shows the efficiency metrics, as a function of DAS (as a measure of DAS target, see Section Methods), for all images and simulation parameters. In each panel, there are 20 stripe-like sets of dots, which represent the 20 distinct natural images (color coded in the left panel). The distinct dots for each stripe represent the parameter variations of the biomimetic model.

The total number of spikes counted from V1 excitatory neurons had a positive link (Pearson correlation coefficient = 0.88, *p* < 0.01) to DAS (Figure 7A, left column). Thus, with biological neurons our network model would have used less energy, when the spatial pattern of V1 firing rates was close to the area summation target.

Our second measure of efficiency, entropy per spike, measures the number of different possible states in the firing rate code, and thus how much information can be coded into the spike train. The highest efficiency emerges when the full range of firing rates is associated with a small mean firing rate. Figure 7B (left column) shows the relationship between entropy per spike of the simulated response patterns and DAS. Our results show a significant relationship (Pearson correlation coefficient = −0.59, *p* < 0.01) between entropy per spike and DAS. Efficiency is high close to target pattern (low DAS), as the highest entropy per spike values coincide with small DAS.

Figure 7C (left column) shows the results for our third efficiency measure, neural population sparseness in relation to DAS. The high population sparseness is associated with the most similar output patterns to the area summation target (Pearson correlation coefficient = −0.56, *p* < 0.01).

All the aforementioned measures together show that efficiency of the simulated response patterns is correlated with the natural spatial structure of the receptive field. There is a clear tendency for parameter combinations producing ASF-type response patterns to be most efficient, although the relation was generally nonlinear and varied between images.

To see how much of the variations between the natural images could be explained by spatial frequencies, we divided the input stimuli in two groups, comprising of images with large proportion of low frequencies and images with a more even distribution of spatial frequencies (Figure 4E). The middle column of Figure 7 is exactly the same as the left column but color coded with either black (for low frequencies) or blue color (for mixed frequencies), for different groups of natural images. The relationship between total number of spikes and DAS is significantly stronger for the low spatial frequencies group (Pearson correlation coefficient = 0.96, compared to 0.81; Wilcoxon rank sum test, *p* < 0.01; here the different natural images are the samples of the Wilcoxon rank sum test). Figure 7B (middle column) shows that the low spatial frequency images have a stronger association between entropy per spike and DAS than the mixed frequency images (Pearson correlation coefficient = −0.71, compared to −0.46; *p* < 0.01). Moreover, the neural population sparseness is also more strongly linked to DAS in the lower than in the mixed frequency group (Pearson correlation coefficient = −0.67, compared to −0.44; *p* < 0.01). The input drive (sum of input spike rate) to the simulator from the images in low frequency group was not significantly different (Friedman, *p* = 0.06) compared to mixed frequency group. These results show that the target ASF in our simulations is associated more strongly with efficient representation for the images containing primarily low frequency information.

In Figure 7A right column, the activation patterns which have very high or very low number of spikes (15% from top and bottom of the dynamic range of the total number of spikes; blue and yellow data points respectively) are color-coded so that the mid-range data can be inspected separately. In this way, we aimed to avoid the parameter combinations that lead to saturated or low-responding activation patterns. Excluding these extreme activation patterns from the analysis, the relationship between the total number of spikes and DAS remained similar to the full data (Pearson correlation coefficient over different images = 0.87, compared to 0.88; Wilcoxon rank sum test, *p* < 0.05). However, the black data points show a stronger association between entropy per spike and DAS (Figure 7B, correlation coefficient = −0.76, compared to −0.59; *p* < 0.001). For the neural population sparseness, the middle part of firing rates dynamic rang showed a reversed link to DAS (black data points in Figure 7C, correlation coefficient = 0.49 compared to −0.56; *p* < 0.001 for the difference). These results show that for the firing rate and entropy per spike association between ASF and efficiency was best when firing rates are not saturated or very low.

**Figure 8:**
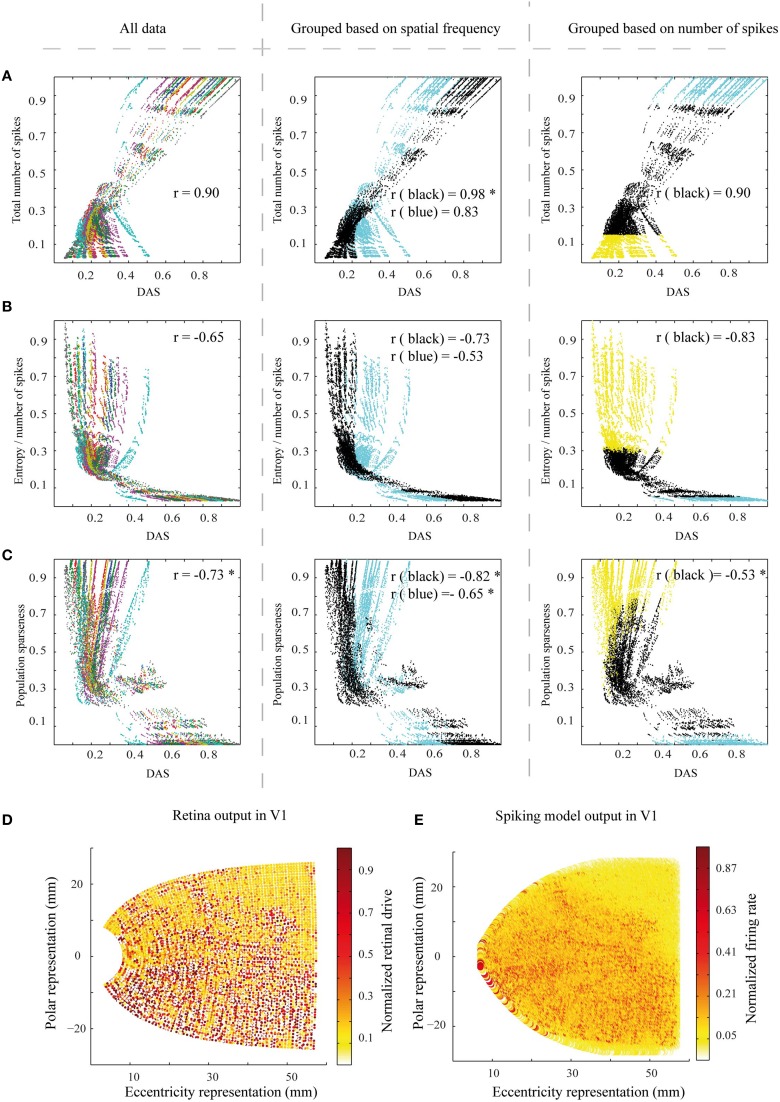
**The efficiency measures for a set of control simulations with a lower resolution of the retina filter**. **(A–C)** The three efficiency measures as a function of DAS, as in Figures 7A–C. The color coding in the columns is the same as in Figures 7A–C. The correlation values differing significantly (Wilcoxon rank sum test with *p* < 0.05) from the original ones at Figure 7 are marked with ^*^. **(D)** The low resolution retina function output for the Figure 2, comparable to Figure 4F. **(E)** A sample of the corresponding simulated output pattern, comparable to Figure 4D. However, note that the color scale is different.

We did two sets of control simulations. In the first set, we checked the effect of retina resolution on the simulations results. The density of midget cells was decreased to 1/5 of the original (see Section Methods). The decrease in density resulted in a lower number of input filters, and correspondingly in spatially larger filters, apparently shifting the filter sensitivity toward lower spatial frequencies. The results show almost similar or slightly better link between DAS and the efficiency measures [Figure 8, all the correlation values which are significantly different from the original ones (Wilcoxon rank sum test with *p* < 0.05) at Figure 7 are marked with ^*^] compared to full-resolution retina.

**Figure 9:**
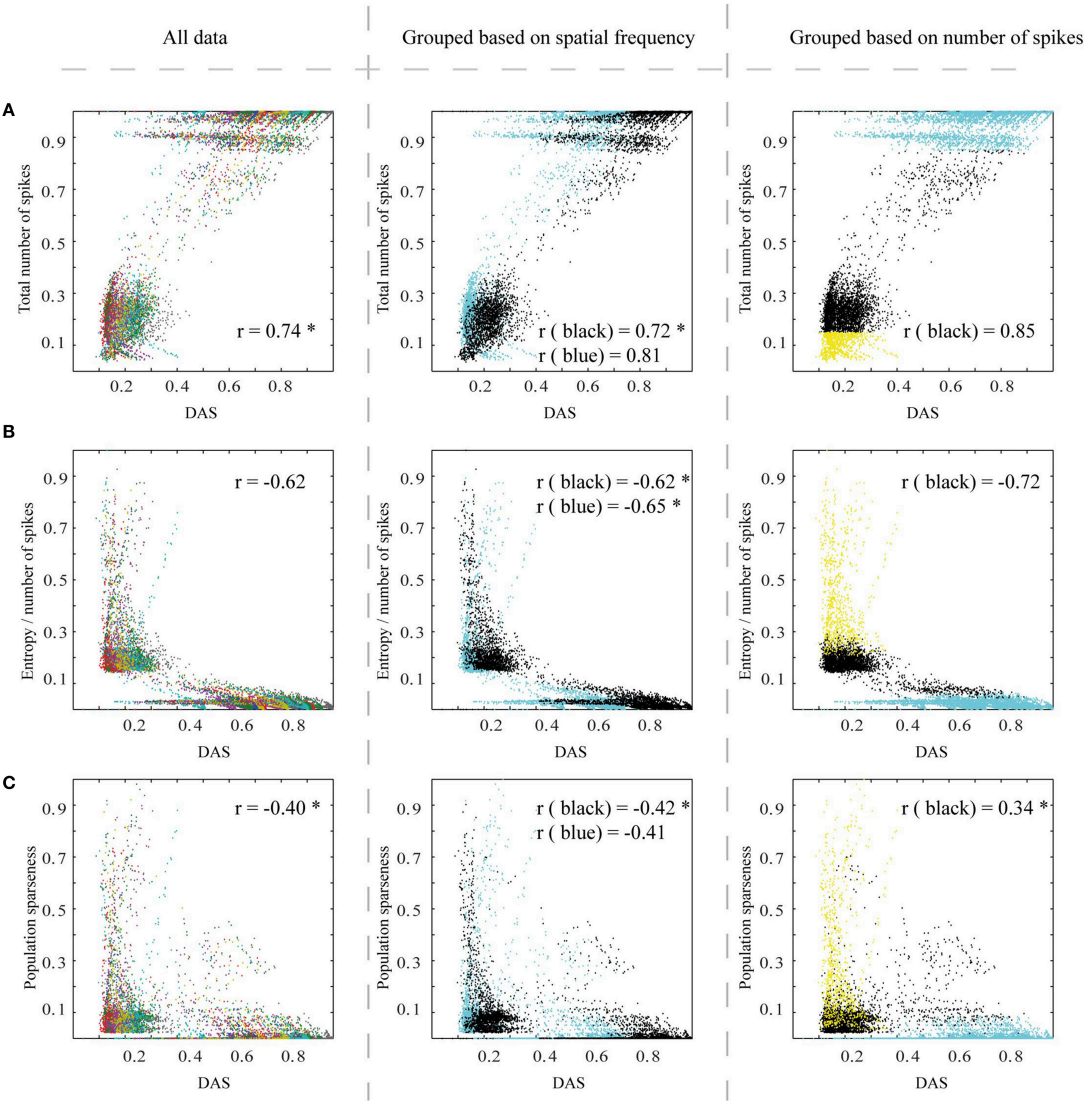
**Efficiency measures for a set of control stimulations with pointwise (instead of compartmental in Figure 7) excitatory pyramidal neurons in the model V1**. **(A–C)** The three efficiency measures as a function of DAS, as in Figures 7A–C. The color coding in the columns is the same as in Figures 7A–C. All the correlation values which are significantly (Wilcoxon rank sum test with *p* < 0.05) different from the original ones at Figure 7 are marked with ^*^. See Figure 7 legend for other details.

In the second set of control simulations, we checked the effect of compartmental neurons (excitatory pyramidal cells at the model V1) on the simulations results. In this control, we replaced the compartmental neurons with pointwise neurons (see Section Methods). The results confirm that the compartments have a clear role in linking the area summation with the efficiency of the network (Figure 9, all the correlation values which are significantly different from the original ones (Wilcoxon rank sum test with *p* < 0.05) at Figure 7 are marked with ^*^).

**Figure 10:**
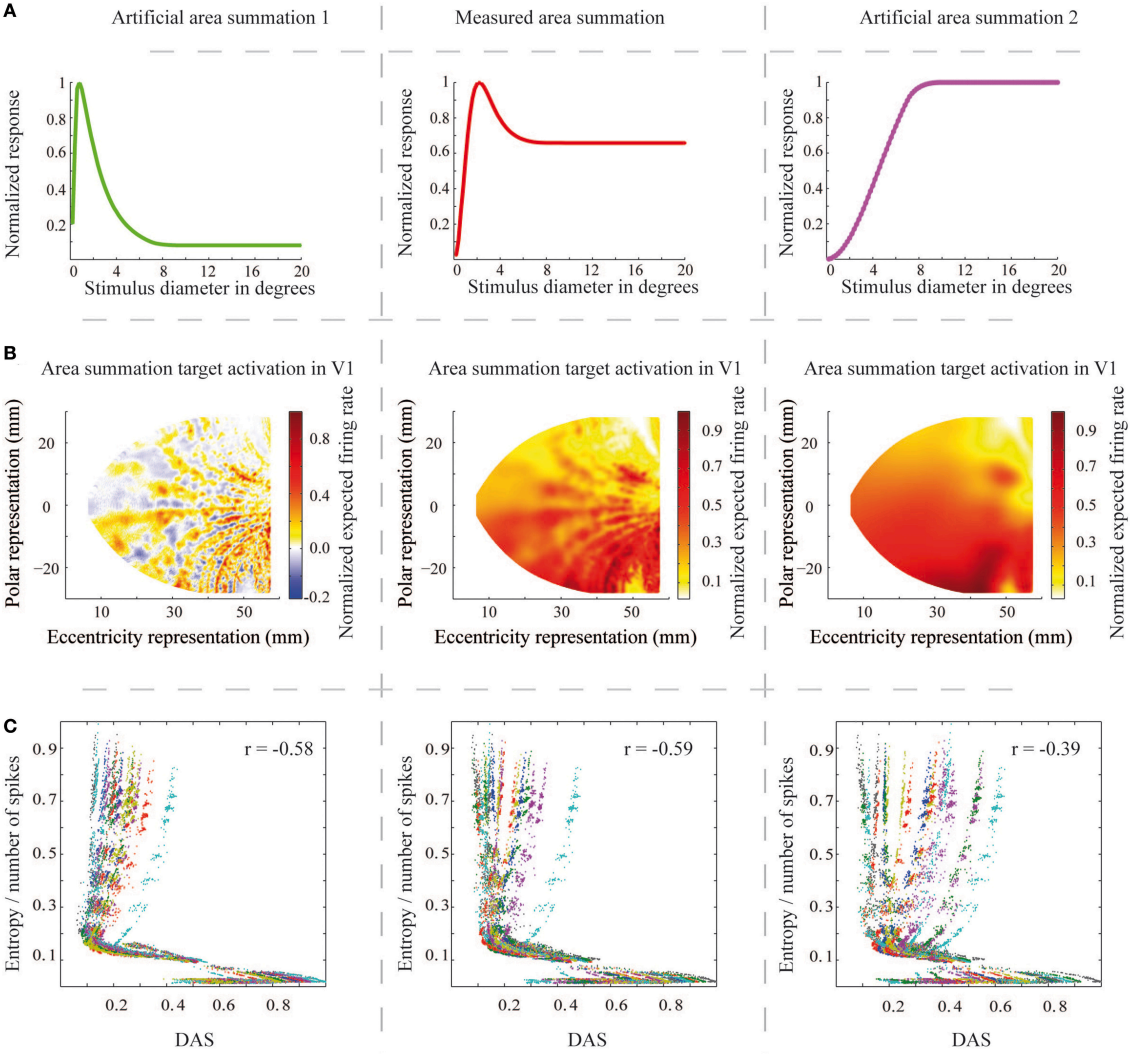
**The area summation functions, the corresponding V1 output patterns and the entropy per spike measures for the natural and two artificial area summation functions**. **(A)** The middle column shows the natural mean area summation function at 14° eccentricity (red color, the same as Figure 1) and the left and right columns show the two artificially created area summation functions at the same eccentricity (green and magenta colors). **(B)** The estimated area summation target patterns for each area summation function for one a sample input natural image (the same image as in Figure 4A). Columns as in **(A)**. **(C)** Relationship between the entropy per spike and DAS (the same as Figure 7B) for each area summation functions. r corresponds to correlation coefficient.

Finally, we tested dependence of the relation between efficiency of the network output and ASF, and the properties of the ASF. We generated two artificial ASFs, to test how the amount of suppression and size of the summation field is related to the network efficiency. If the natural ASF is optimal for natural images processed by a biophysically meaningful network, we should observe loss of efficiency when changing the function form. Figure 10A shows the three function forms (the middle column contains the natural ASF) and Figure 10B the V1 area summation target pattern for the sample image of Figure 4A, estimated for each ASF. Figure 10C shows the entropy per spike vs. DAS for the ASF in the corresponding column, with the middle panel thus reproducing the data of Figure 7B. The results show that, with the larger summation field and no suppression, the entropy per spike of the activation patterns is less relevant to the similarity to predicted area summation pattern than with the natural ASF (Pearson correlation coefficient = −0.39, compared to −0.59; Wilcoxon rank sum test, *p* < 0.001). However, the smaller summation field and stronger suppression showed almost similar dependency between entropy per spike and DAS than natural ASF (Pearson correlation coefficient = −0.58, compared to −0.59; *p* = 0.65).

## Discussion

We studied the relationship between the ASF and the efficiency of neural population code, using a biomimetic simulation of the visual cortex. Our results show that the ASF is associated with reduced energy consumption, better information carrying capacity (as measured by the entropy per spike), and higher population sparseness. The relation was typically nonlinear, but consistently present. Consequently, we suggest that the nonlinear and nonmonotonic area summation property of visual neurons is related to energy efficiency in the visual system. The result was further refined in several control setups. Our simulator seemed to work better with low spatial frequency input than with the images containing both high and low frequencies suggesting need to refine the model in future studies. In addition, excluding the parameter combinations which led to saturated or low-responsive activation patterns improved the link between entropy per spike and ASF, suggesting that the association is best at the dynamic range of firing rates. Moreover, an artificial ASF with a monotonically increasing summation function was more poorly associated with the efficiency, compared to the natural ASF of macaque monkeys. This indicates that the surround suppression of the ASF is important for the efficiency.

Our findings are in line with biological data, such as a previous contextual modulation study (Vinje and Gallant, 2000), which showed a link between stimulation outside the CRF and sparse code representation of the visual input. Moreover, Schwartz and Simoncelli (2001) suggested that the visual system reduces dependencies between single cells and thus increases efficiency by means of the knowledge of image statistics surrounding the CRF. Our results are also in line with an association between contextual modulation and efficient coding of the fMRI responses (Sharifian et al., 2013).

In contrast to the experimental studies *in vivo*, the biomimetic neural network simulation approach gives us a unique tool to quantitatively characterize this efficiency. In particular, we found a clear association between horizontal inhibitory connectivity and efficiency. This can be generalized to horizontal connectivity including the excitatory to excitatoryconnections, because their relative contribution increases when the excitatory to inhibitoryconnection strengths decrease. Interestingly, the minimal DAS is not at the most efficient point but somewhat offset from this point. We assume that the lowest firing rates (on average 13 Hz firing rate for the last three columns of Figure 6B) are too small to carry significant information, and thus the ASF *in vivo* is offset from this end.

Recently, Nurminen and Angelucci (2014) claimed that horizontal connections have a specific role in network efficiency. The horizontal connections carry information about the orientation preference, and this information might be essential to reduce the statistical redundancy in cortical representation of spatially neighboring edges (Geisler et al., 2001). In line with this statistical dependency, the near surround is more strongly orientation tuned than the far surround in both human psychophysics and monkey single cells (Shushruth et al., 2013). Our model has no orientation preference and thus no optimization target for coding images efficiently. In other words, we did not maximize the efficiency for the information content of the images; instead we only compared the ASF spatial structure and efficiency.

While the feedback strength did not have much effect on efficiency, the connectivity structure of the feedback did. We used a compartmental neuron model for the V1 excitatory pyramidal neurons. Heikkinen et al. (2015) emphasized the role of passive dendritic properties (Rall, 1962) of pyramidal neurons for a good simultaneous simulation of both spiking networks and experimental fMRI data. In particular, the much smaller spread of target ASF prediction in their study could not be reached with concurrent much larger spread of the fMRI signals, without a compartmental neuron model. We found a similar result with efficiency. Compared to simulations with a soma-only model, the model with compartmental neurons presented a stronger association between ASF and the efficiency of the neural network (Figure 9).

In summary, we suggest that the basic low-level connectivity plan in visual cortex, resulting in ASF-like spatial receptive field structure, is related to the evolutionary pressure for coding efficiency. Our second efficiency measure (entropy per spike) is a measure of energy efficiency, but not coding efficiency, because it does not necessarily lead to better classification of the activation patterns according to their information content. A network that is efficient in energy consumption might have no useful information in the network to classify images. In our model network, the lack of information content is expected because in our neural network the receptive fields have no structure, just the position, and scatter. Future work will need to address the effect of receptive field structure (e.g., orientation preference) and meaningful information carried by the network on the model output distance to ASF.

Our model is a rough simplification of the primate visual system, and the reduction of complexity most likely modifies the results of this study. First of all we have a reduced number of units in our neural network model, compared to the primate visual cortex, with our resolution corresponding to ~0.1° at 5° eccentricity. This may directly impact the processing of the high-frequency content of the images. Further, the model implements only one higher visual area and ignores corticothalamic feedback. Second, our simulation of visual cortex lacks the layered structure of the primate cerebral cortex. Instead, we model all neural activation with one layer, which most likely results in important omissions of parameters affecting the results. Third, we only have spatial position tuning in our model and no orientation, speed, disparity, spatial frequency, ocular dominance or wavelength tuning, as primate V1 cells do. One discrepancy with primate cortex is apparent in Figure 6A (solid blue curve). Whereas, the general ASF form (dashed curve, monkey data) could be reached with the biomimetic model simulation, the size of the summation field was on average smaller than in monkeys (as exemplified in Figure 1) at this eccentricity. Other example cells at different eccentricities showed similarly too small summation field sizes. Mammal cerebral cortex hosts several types of inhibitory neurons with distinct anatomical and physiological characteristics (Ascoli et al., 2008). Discrepancy in the summation field size can easily emerge from omission of this diversity, and remains a challenge for future studies. Including different types of inhibitory cells with distinct inhibitory field size will probably enhance our model to better fit the natural summation field size. Such enhancement of the model is not expected to ruin the relationship between ASF and our efficiency measures, but underlies the qualitative nature of our result. For example in Figure 10, almost similar (Pearson correlation coefficient = −0.58, compared to −0.59; *p* = 0.65) relationship between ASF and the efficiency measures emerge with smaller summation field and stronger suppression. In addition, the result of Figure 10 shows weaker relationship between ASF and the efficacy measures in absence of inhibitory surround (Pearson correlation coefficient = −0.39, compared to −0.59; Wilcoxon rank sum test, *p* < 0.001), which suggests that inhibition rather than summation field size is the important attribute of the ASF in maintaining efficacy of the system. Nevertheless, our model shows robust dependence of efficiency with the non-linear ASF, and the mismatch in the summation field size most likely creating a positive baseline to the total error measure. Moreover, our results show considerable variations in the absolute values of the efficiency measures for a specific DAS value which might emerge from un-physiological parameter ranges, non-linearities of the system, or the unavoidable simplifications in our model.

We run the simulation for 200 ms before the visual input to reach a stable baseline and thereafter presented stationary input for 300 ms. The mean firing rate was calculated based on the last 300 ms time interval.

Primarily we used comparable density of midget cells to the human retina in our simulations. This results in a very high computational load, which can be suboptimal given the much lower sampling of the biomimetic cortex. A set of control simulations showed that 1/5 of this density of midget cells can lead to a somewhat better association between area summation and efficiency of the neural network (Figure 8). The better link between DAS and efficiency measures in some panels may emerge from high-spatial frequency noise in the simulation output with the high-resolution retina filter. The high input resolution (compare Figure 4F with Figure 8D) leads to a V1 activation pattern with higher spatial frequencies (compare Figure 4D with Figure 8E). If the spatial frequencies in the biomimetic simulations are higher than after filtering the image with the model ASF, the residual firing patterns for the highest frequencies would show up as additional noise.

Clearly, the optimal size for the summation and suppression fields of the ASF depends on the input features. Each 20 gray-scale natural images drew a distinct path into the three diagrams of efficiency vs. DAS. The results from middle column of Figure 7 show that the simulated network output associates best with the efficiency measures for the group of images containing mainly low frequency information than for the group of images containing both high and low frequency information. In calculations of the target ASF, as a first approximation, we used the average values of the measured summation field, surround diameter and suppression levels at each eccentricity and ignored the diversity of these values in our model (Figure 3). This might explain why the results show variation in efficiency vs. DAS between different stimuli (Figure 7, middle column). The variability of the receptive field parameters in the real cortical neurons may help capture the range of spatial frequencies present in natural images. Alternatively, lack of biological diversity in the inhibitory neuron population or other simplifications in the model structure may explain the less accurate fit of the model output with efficiency, when the stimuli comprised the higher spatial frequencies.

For each natural image, we had 625 simulation runs, which came from varying the extrastriate connectivity and inhibition within V1 as free parameters. In the simulations, the number of lateral connection between V1 excitatory and inhibitory cells seems to be a more determinative parameter to match the target ASF compared to the feedforward-feedback connections between V1 and the extrastriate area (Figure 6B). Clearly some of these combinations lead to saturated or nearly non-responding activation patterns (Figure 7A, right columns). In addition, these data points caused huge variation in absolute value of our efficiency measures for a specific DAS value. The link between efficiency measures and DAS was much clearer in the case of excluding these extremes from our analysis (Figure 7, right column). The selection of the outlier threshold is always somewhat arbitrary. The main finding from this analysis is a well matched relationship between entropy per spike and ASF with reasonably physiological firing rates (on average 14–78 spikes per neuron per 300 ms).

The ASF can be regarded as a comprehensive model of the spatial receptive field, including the summation close to the center of the receptive field (CRF) as well as contextual positive (summation field) and negative (surround suppression) modulation of a neuron's response. In the current study, we were interested in the relation of the ASF and the efficiency of neural output at the system level. Apparently, the nonlinear ASF helps to avoid saturations of the response patterns. The largest spike counts were associated with large distances from the predicted ASF target. In addition, the middle 70% of the dynamic range of the firing rate showed strong association between entropy per spike and ASF. While the relation between sparseness and the ASF target was not so straightforward within this same dynamic range and indeed appeared to be positively correlated, high sparseness were overall well associated with ASF. Moreover, for the low-resolution input, sparseness had an overall better association with the ASF.

In summary, our study shows that the nonlinear ASF, which is a mathematical formulation of extra-CRF modulation onto the CRF responses, is related to efficiency of a neuronal population model.

## Author contributions

FS designed, simulated, and analyzed the neural network and wrote the paper. HH designed the neural network, and participated in writing the paper. RV participated in data analysis and writing the paper. SV designed and analyzed the neural network, and wrote the paper.

### Conflict of interest statement

The authors declare that the research was conducted in the absence of any commercial or financial relationships that could be construed as a potential conflict of interest.
